# Identification and validation of ubiquitin-proteasome system related genes as a prognostic signature for papillary renal cell carcinoma

**DOI:** 10.18632/aging.204383

**Published:** 2022-11-16

**Authors:** Xin Zhang, Xinli Liu, Renhua Xiong, Han-Xiang An

**Affiliations:** 1 Department of Medical Oncology, Xiang'an Hospital of Xiamen University, Fujian 361005, China; 2 Xiamen Key Laboratory of Endocrine-Related Cancer Precision Medicine, Fujian 361102, China

**Keywords:** ubiquitin-proteasome system genes, risk model, PRCC, TCGA, bioinformatics, prognosis, immunity

## Abstract

Abstract: Dysregulation of the ubiquitin-proteasome system (UPS) pathway greatly affects uncontrolled proliferation, genomic instability, and carcinogenesis, particularly in those with renal papillary cell carcinoma (PRCC). However, there is little information at the molecular level about the full link between changes in the genes involved in ubiquitin-mediated proteolysis and PRCC.

Methods: The Cancer Genome Atlas (TCGA) and GeneCards databases were utilized to find the clinical data and gene expression patterns of patients with PRCC. Univariate Cox regression analysis and absolute shrinkage and selection operator (LASSO) analyses identified a risk signature formed by ten optimal UPS genes. The predictive value of the risk signature in TCGA-PRCC cohorts was evaluated using Kaplan-Meier analysis and receiver operating characteristic (ROC) curves. By utilizing GO enrichment and the KEGG pathway, the interactions of differentially expressed genes connected to ubiquitin-mediated proteolysis were functionally examined. The protein expression of the hub genes was affirmed using the Human Protein Atlas (HPA) database. The effectiveness of particular CDC20 and UBE2C *in vitro* was confirmed by experimental research.

Results: Ten of the best ubiquitin-mediated proteolysis genes (UBE2C, DDB2, CBLC, BIRC3, PRKN, UBE2O, SIAH1, SKP2, UBC, and CDC20) were detected to create a risk signature. The high-risk score group stratified was associated with advanced tumor status and poor survival of PRCC patients. 10 genes were also found to be associated with the cell cycle pathway and ubiquitin-mediated proteolysis to GO and KEGG analysis. Of these 10 genes, CDC20 and UBE2C are highly expressed in tumor tissue and correlated with cancer immunity founded on the analyses of the expression of human protein atlas and TISIDB. The downregulation of UBE2C facilitated tumor inhibition and the anti-immune effect was confirmed by *in vitro* experiments.

Conclusion: Our results indicate that the risk model created from the ubiquitin-mediated proteolysis genes can be reliably and accurately predict the prognosis of PRCC patients, highlighting its targeted value for PRCC treatment. Particularly, the expression of UBE2C may be crucial for the prognosis and immunological treatment of renal cancer.

## INTRODUCTION

Renal papillary cell carcinoma (PRCC) represents about 15% of all renal cell carcinoma, which is the most common type of kidney cancer [1–3]. Despite multimodal conventional therapy, including surgical resection and radiotherapy, advanced and metastatic kidney cancers remain a major cause of death in human cancer [4]. Within decades, the amount of research on molecular markers and multitargeted drugs has produced a limited role in prolonging the life expectancies of advanced and metastatic kidney cancer patients.

Driven by the success of immune checkpoint blockade applied in many cancers, research on the immunotherapy of kidneys had increased exponentially in the past few years. The PD-1/PD-L1 axis and the CTLA-4 receptor are the most common immune checkpoint in advanced and metastatic PRCCs [5, 6]. However, most renal cancers have been proven to be refractory to current immuno-therapies [7]. This has raised our interest in finding molecular mechanisms that deeply regulate response to immunotherapy in PRCC. Previous studies investigated potential biomarkers for the clinical response of immunotherapies in PRCC by using bioinformatic methods. Xi et al. [8] analyzed and compared the differentially expressed genes of PRCC obtained from the Gene Expression Omnibus (GEO) databases and found high expression levels of PTTG1 significantly associated with tumor microenvironment including lymphocytes, immune modulators, and chemokines. Yutao et al. [9] constructed a three-gene risk model for predicting prognosis in patients with PRCC from TCGA and three GEO series (GSE) datasets.

Ubiquitination, which mostly consists of E1, E2, and E3, is one of the most significant post-translational modifications [10–12]. Dysregulation of ubiquitination has been observed in various types of cancers, including PRCC [13–16]. By way of illustration, TRIM37 is a novel discovered E3 ubiquitin ligase and can promote RCC cell EMT and malignant progression via TGF-β1 signaling activation [17]. TRIM13, a RING domain containing E3 ubiquitin ligase, may serve as a candidate for RCC prognostic marker and potential therapeutic target by decreasing the RCC metastasis and invasion ability [18]. These studies have revealed a connection between the development of PRCC and changes in certain genes relevant to ubiquitin-mediated proteolysis. However, no research has yet thoroughly examined alterations of the UPS ubiquitin-mediated proteolysis process in the carcinogenesis and development of PRCC.

In this study, by analyzing the dataset from the TCGA database, we aim to systematically study and verify the expression characteristics of ubiquitin-proteasome system-related genes in PRCC. We identify a number of ubiquitination-related genes that were strongly correlated with PRCC patients’ prognoses using a variety of statistical techniques. Finally, based on the screened risk genes, we developed a new and accurate risk model to forecast the prognosis of PRCC patients.

## MATERIALS AND METHODS

### The cancer genome atlas cohort

The TCGA (https://portal.gdc.cancer.gov/) and cbioportal (http://www.cbioportal.org) databases were used to gather the molecular mRNA seq data and clinical information of 539 PRCC patients with 72 normal samples. The pathological characteristics of the tumors and clinical features of these patients with PRCC are all included in the data cohort.

### Identification of prognostic UPS genes for the construction of a risk model

Ubiquitination-related genes selected based on the Genecard database that was substantially correlated with the prognosis of PRCC patients were filtered out using univariate Cox regression (*P* < 0.05). These ubiquitination-related genes chosen by univariate Cox regression were analyzed using the Least Absolute Shrinkage and Selection Operator (LASSO) algorithm with the “glmnet” R package [19]. Selected candidate UPS genes were finally obtained to make the risk model in PRCC. All *p*-values were lower than 0.05.

### Construction of the prognostic risk model in the PRCC cohort

After enrolling into the LASSO algorithm, hub genes were screened founded on the minimum criteria. The risk score of each patient was estimated according to the expression levels of mRNA and estimated regression coefficients of the hub genes. The coefficients of the hub genes were obtained from the Cox proportional hazards regression analysis. The formula was given as follows: RiskScore (RS) = (expression of mRNA1 × Coefficient of mRNA1) + (expression of mRNA2 × Coefficient of mRNA2) + (expression of mRNAn × Coefficient mRNAn). We determined each patient’s risk score, depending on the coefficient and expression level of the ten genes. Cancer patients were separate into two risk score subgroups, each representing the high and low RS group founded on the median value of the RS by using the “Rtsne” R package. T-Distributed Stochastic Neighbor Embedding (t-SNE) was performed to explore the distribution of different risk groups. The low-risk group gets a better prognosis.

### Evaluating the prognostic value of the gene signature

To validate the stability and reliability of the risk model, multivariate Cox analysis with LASSO and Kaplan Meier analysis with the log-rank test was utilized. Receiver operating curves (ROC) and calculated the area under the curve (AUC) were developed to ascertain if the current model can accurately predict the overall survival (OS) of PRPP patients. A C-index value of 0.75 or above was deemed to have strong predictive power, and a value of more than 0.6 was deemed adequate for survival predictions [20]. We considered whether UBE2C has an impact on the prognosis and immune-related genes in PRCC. Information was mined regarding the clinicopathological features, KM curve, and correlation between UBE2C and CDC20 from the UALCAN portal (http://ualcan.path.uab.edu/).

### Comprehensive and enrichment analysis of protein-protein interaction

A large database of consolidated and integrated information about protein-protein interactions was found on the public website Search Tool for the Retrieval of Interacting Genes/Proteins (STRING). Network data of selected top 20 protein-protein interactions (PPI) was obtained after importing those hub genes into the STRING database (https://string-db.org/). Gene set enrichment analysis (GSEA) was carried out on twenty interacting genes and ten hub genes in the TCGA cohorts to find enriched pathways linked to risk models. Based on the R package “clusterProfiler,” the study was shown using “ggplot2” (|log2FC| ≥ 1, FDR < 0.05).

### The human protein atlas and statistical analysis

Human Protein Atlas (https://www.proteinatlas.org) is an online database that enables the mapping of all human proteins in cells, tissues, and organs utilizing antibody-based imaging, mass spectrometry-based proteomics, transcriptomics, and systems biology. Utilizing the website, we compared the level of gene transcription to the level of protein expression for the CDC20 and UBE2C.

GraphPad Prism (Version 7.0) and ImageJ (V. 1.8.0.112) were used to create figures and conduct statistical analysis. The expression of hub genes in various groups was compared using one-way ANOVA, unpaired or paired *t*-test, and Dunnett's test. The Chi-square test, fisher's exact test, and logistic regression was performed to analyze the correlation of hub genes expression with clinicopathological factors and immune infiltration around tumors.

### Correlation between mRNA expression and immunity

**Figure 1:**
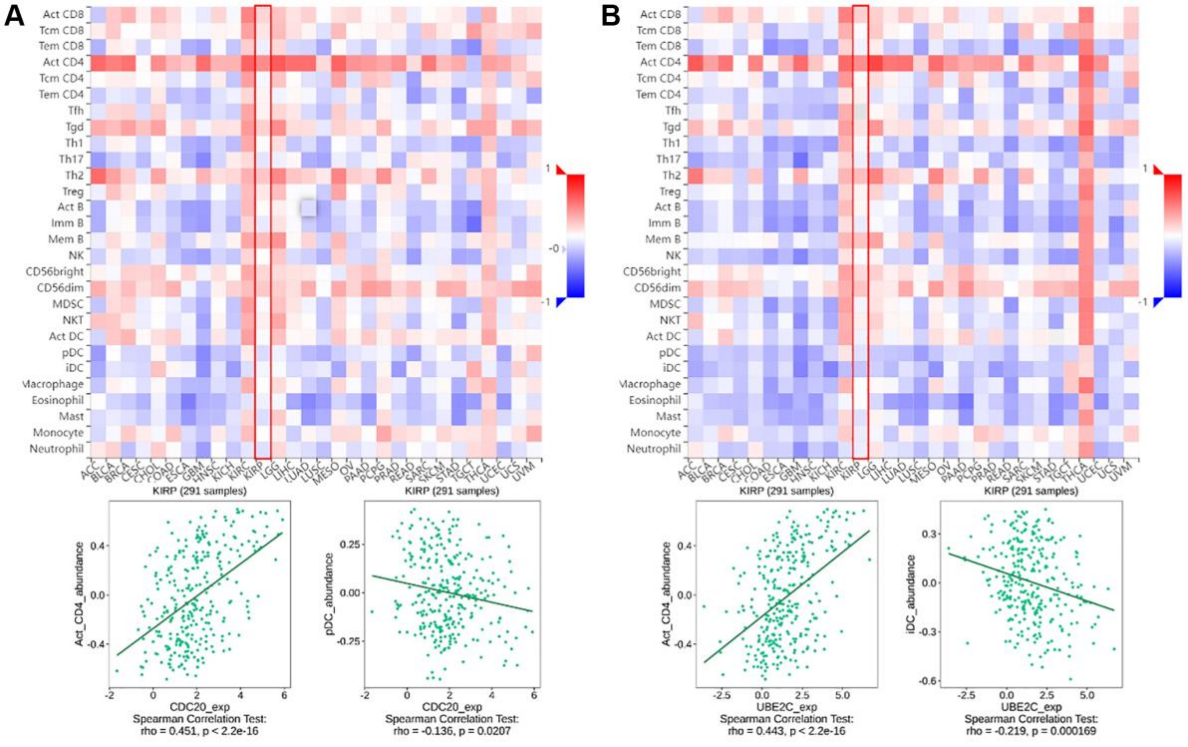
**The relationship between CDC20 and UBE2C and immune infiltration levels in PRCC is based on the TISIDB website**. (**A**) Heatmap of the relationship between CDC20 and abundance of the 28 immune infiltration lymphocytes in PRCC. The two highest positive and negative lymphocytes correlation with CDC20 expression are Act_CD4 T cell and pDC in PRCC patients. (**B**) Heatmap of the relationship between CDC20 and abundance of the 28 immune infiltration lymphocytes in PRCC. The two highest positive lymphocytes and negative lymphocytes correlated with UBE2C expression are Act_CD4 T cell and iDC in PRCC patients. “Red” represents a positive correlation and “Blue” represents a negative correlation. ^*^P < 0.05, ^**^P < 0.01, ^***^P < 0.001.

**Figure 2:**
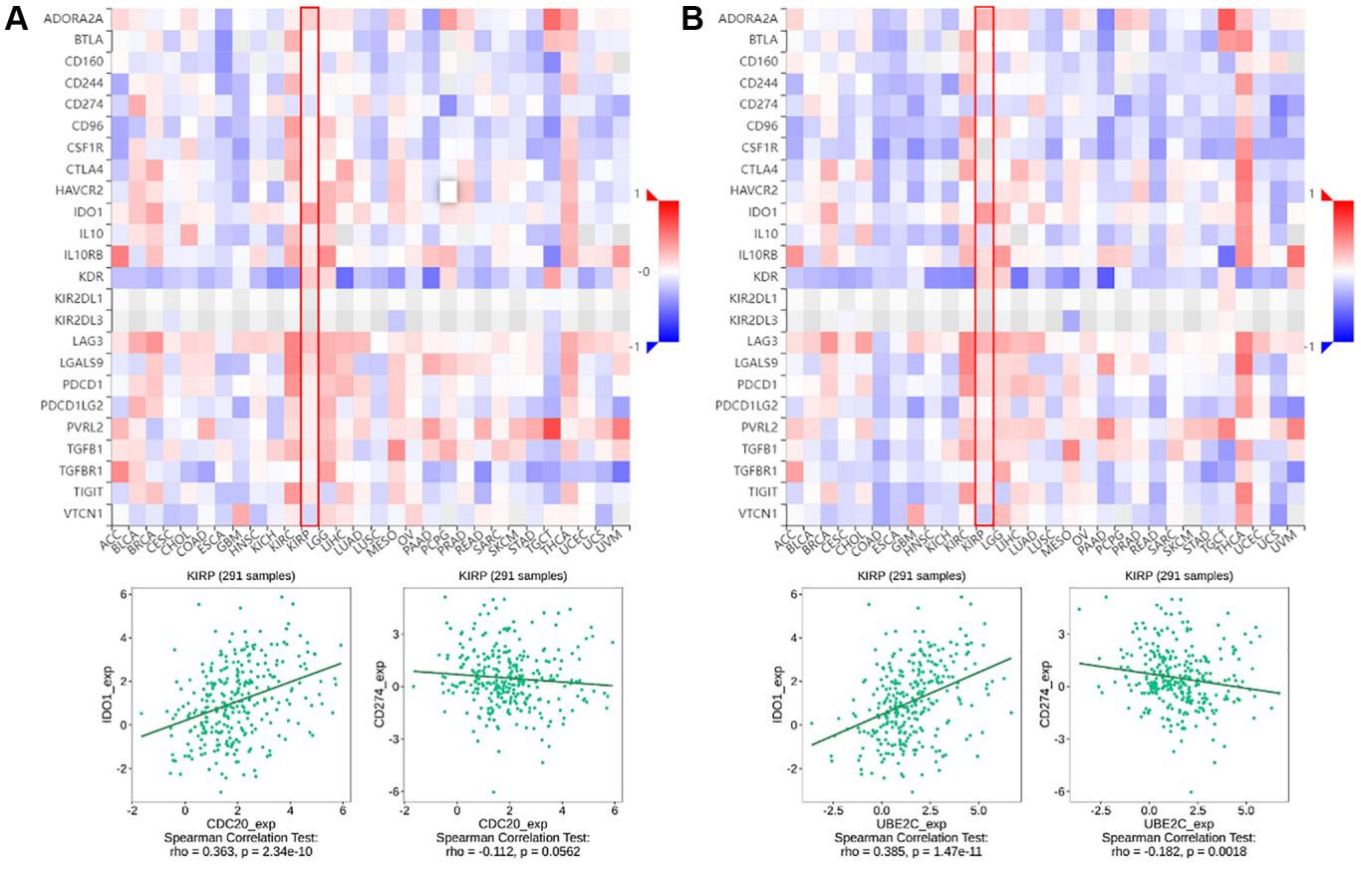
**The relationship of CDC20 and UBE2C with immunoinhibitors in PRCC patients is based on the TISIDB website.** (**A**) Heatmap of the relationship between CDC20 expression and abundance of the 24 immunoinhibitors. The two highest immunoinhibitors correlated with CDC20 expression are IDO1 and CDC274 in PRCC patients. (**B**) Heatmap of the relationship between UBE2C expression and abundance of the 24 immunoinhibitors. The two highest immunoinhibitor correlated with UBE2C expression are IDO1 and CDC274 in PRCC patients. “Red” represents the positive correlation and “Blue” represents the negative correlation. ^*^P < 0.05, ^**^P < 0.01, ^***^P < 0.001.

TISIDB (http://cis.hku.hk/TISIDB) is a web server for comprehensive analysis of tumor-infiltrating immune cells, and immunoinhibitor, etc. The abundance of immune infiltrate cells and immunoinhibitor can be estimated from gene expression profiles as described (Figures 1 and 2).

### Cell line and cell culture

The human RCC cell line 769-P was acquired from the Chinese Academy of Sciences and cultured in RPMI 1640 medium supplemented with 10% FBS and 1% penicillin-streptomycin. Cells were incubated at 37°C in a humidified atmosphere of 5% CO2.

### Cell viability and proliferation assay

Cell proliferation was calculated by MTT assay. The medium with the different groups was removed and cells were incubated with 20 μl MTT (5 mg/mL) for 4 h at 37°C. The optical density was recorded at 490 nm using a Microplate Reader (Thermo Scientific, USA) after dissolving the formazan with volatile DMSO for 10 minutes in a swing bed.

### Migration and invasion assays

Cell migration was assessed using a QCM™ 24-well Cell Migration Assay. 24 h after transfection, cells were trypsinized and seeded in chambers at the density of 5 104 cells per well and cultured with RPMI 1640 medium with 1% serum, while 700 μl of 20% FBS–1640 medium was added to the lower chamber. After 48 h, the bottom of the membrane was fixed to 100% methanol at room temperature for 30 minutes. With cotton swabs, non-migrated cells were removed. The membrane was subsequently 0.5% crystal violet dye for 10 minutes. The image of the cells was caught in the microscope after the membrane had dried at room temperature. Five random fields of view were counted and the relative fold of migration in each group was comparable to the control group.

### siRNA transfection

Silencer siRNA and Negative Control siRNA are directly purchased from RiboBio (Guangzhou, China). Interference sequences are below: siR UBE2C-1, 5′-CACAGACACTCACCTTACT-3′; siR CDC20-1, 5′-GCAGAAACGGCTTCGAAAT-3′. 769-P cell were transfected with siRNA using TurboFect™ Transfection Reagent (Thermo Fisher, USA) in serum free media following the manufacturer’s protocol. Firstly, 2 × 10^5^ cells were seeded in 6 wells in 1640 media complemented with 10% FBS (without antibiotic) overnight. The following day, the media was replaced by 2 mL of 1640 serum free media (SFM). 3 μg of siRNA was mixed with 10 ul transfection reagent in 250 ul 1640 SFM and incubated for 20 min then added to 2 mL 1640 media. After 8 h of incubation, the transfection media was removed from the cells and the cell was incubated for further experiments.

## RESULTS

### Screening UPS genes associated with PRCC prognostic and constructing a risk model

**Figure 3:**
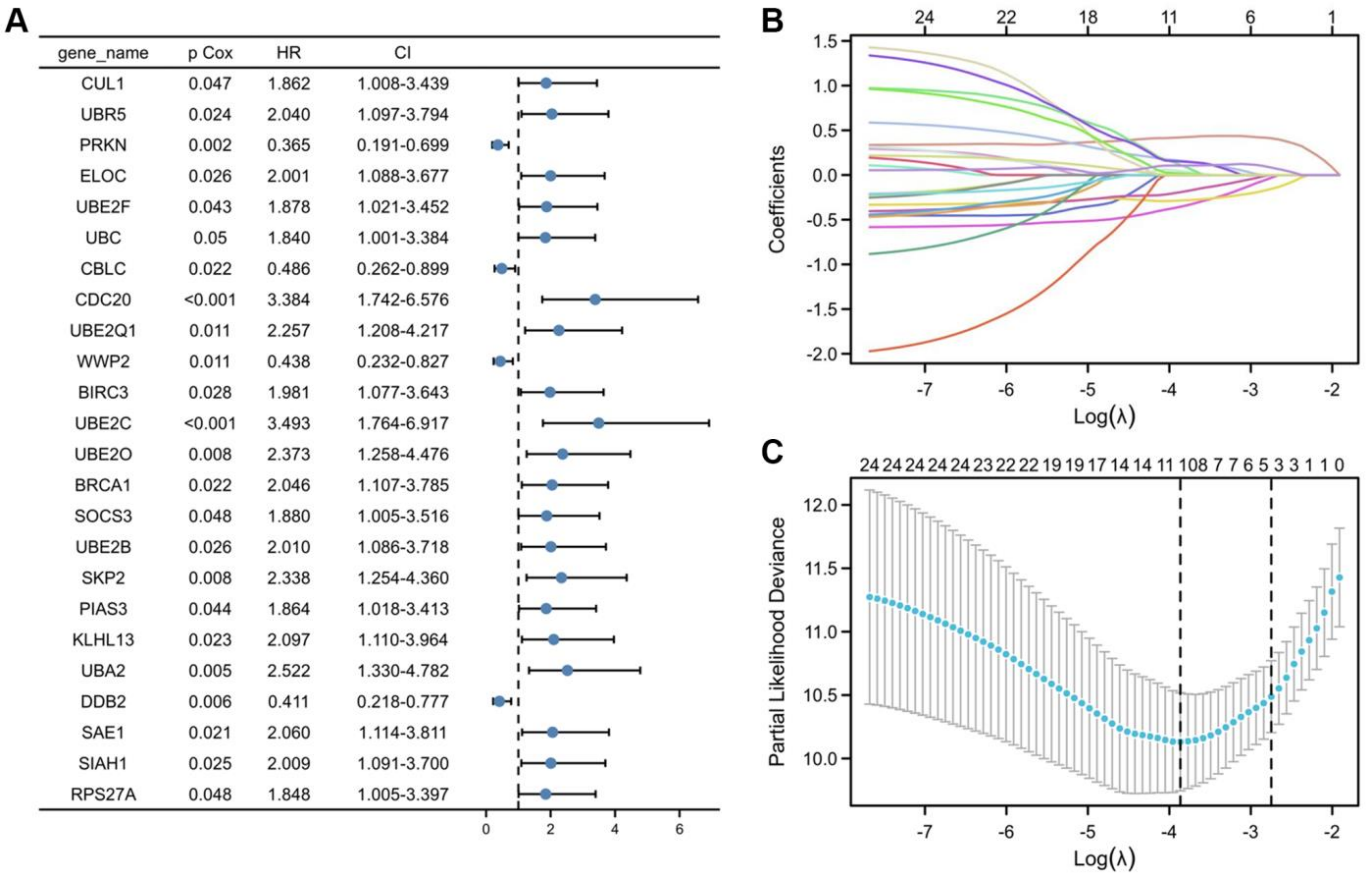
**Selection of UPS genes associated with the survival of PRCC by univariate Cox regression analysis and Lasso regression analysis.** (**A**) Forest plot of Pearson's correlation coefficients between 24 selected genes and patient survival in TCGA-PRCC. Values display in the left-hand column (P Cox). The hazard ratio (HR) is a comparison between the probability of events in cancer, associated with the probability of events in normal tissue. Every coefficient is shown as a point with a 95% confidence interval (CI). Dots represent the mean and CI of the random-effect model estimation; the horizontal line shows the prediction interval. (**B**) Lasso coefficient profiles of the 24 risk genes from univariate Cox regression analysis. The lines stand for the coefficient of Lasso. (**C**) Cross-validation for selecting risk genes for the LASSO model. The number on top of the plot displays the number of genes of each model. The two dotted vertical lines were drawn based on the optimal data according to the minimum criteria (left line) and 1se criteria (right line). The left vertical line represents the 10 genes finally identified. ^*^P < 0.05, ^**^P < 0.01, ^***^P < 0.001.

To create predictive gene signatures, 140 genes involved in ubiquitin-mediated proteolysis were screened in the GeneCards according to their molecular functions and genetic ontology. Using univariate Cox regression analysis, 24 ubiquitin-mediated proteolysis genes were selected, with substantial predictive value (*P* < 0.05) displayed in Figure 3A. The most important genes involved in ubiquitin-mediated proteolysis were subsequently identified using a least absolute shrinkage and selection operator (LASSO) regression model (Figure 3B). The coefficients of these genes in the LASSO penalized Cox regression was decreased as a result of the change in the log λ (a tuning parameter), indicating that they overlooked impacts since they were decreasing parameters. After cross-validation, ten genes met the required minimal partial likelihood of deviation (Figure 3C). Therefore, these 10 ubiquitination-related genes were chosen for the following investigation.

### Validation of the signature in the PRCC cohort

**Figure 4:**
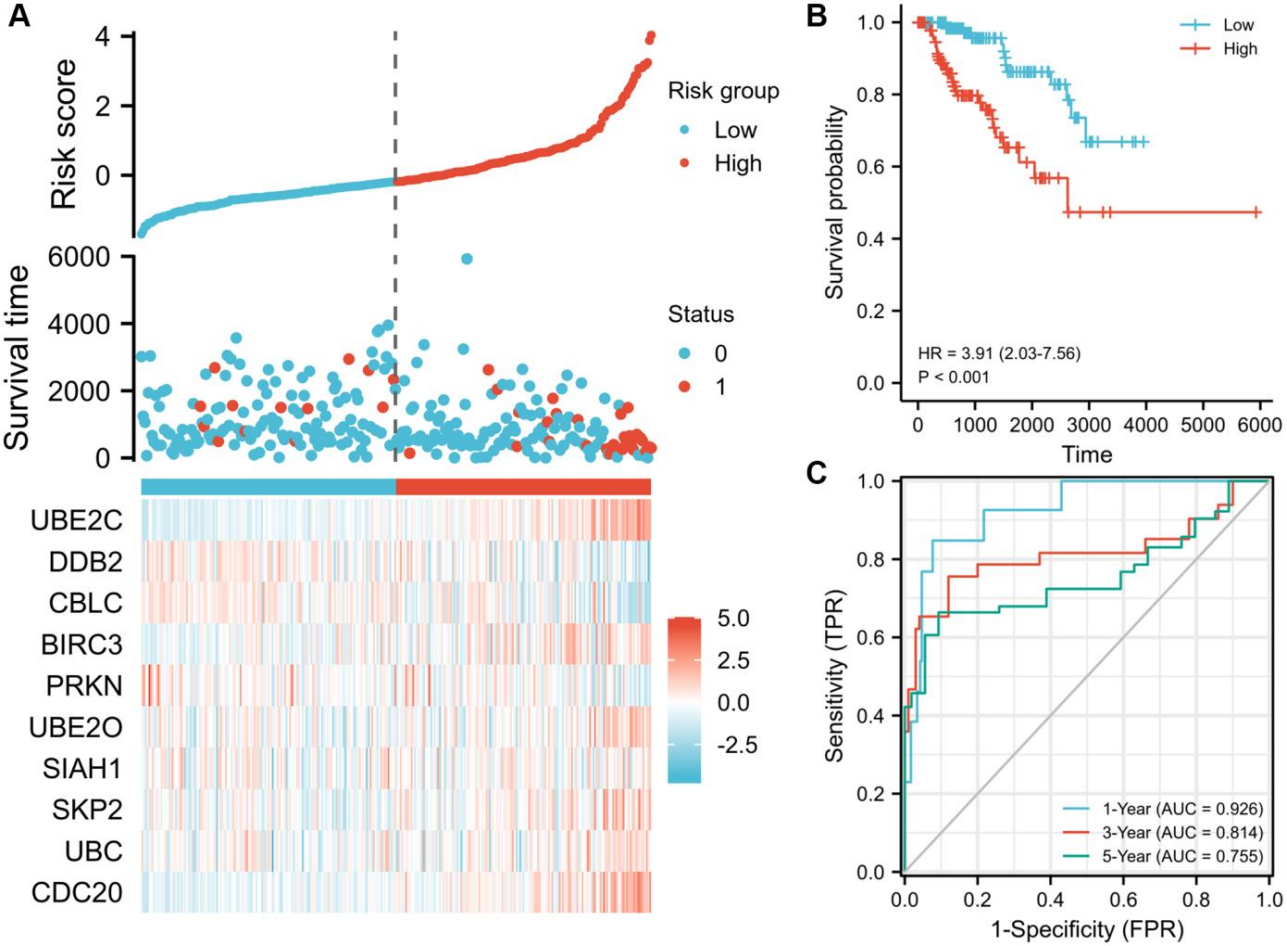
**Prognostic risk score model analysis of risk models in OS of TCGA-PRCC cohort.** (**A**) Scatter plots of risk score (top), scatter plot of survival time distribution (middle), and gene expression heat maps (bottom). (**B**) Kaplan Meier curves for overall survival between low- and high-risk score patients. (**C**) Time-dependent ROC curves for the prognostic model for 1-, 3-, and 5-year OS of PRCC. The AUC values represent the predictive performance of the gene signature and clinical risk factor. Abbreviations: OS: overall survival; AUC: area under the curve; HR: hazard ratio. “Red” represents the positive correlation and “Blue” represents the negative correlation.

Lasso logistic regression analysis was used to determine the best combination of ten genes to use as a predictor of OS in PRCC. Founded on the level of gene expression and coefficients, a risk score formula was developed to assess the prognosis risk score for each patient. The risk score of these patients was ranked and their distribution was analyzed. Based on the median value of the risk score, patients were separate into two groups, the high-risk group and the low-risk group (Figure 4A, top). The survival status of each patient in the PRCC patients was presented in Figure 4A (middle). According to Figure 4A, the higher the risk scores, the shorter the OS of PRCC patients. Consistently, patients with the high-risk score had poorer overall survival than cases of low risk score (Figure 4B, *P* < 0.001), suggesting the prognostic potential of the risk score for PRCC patients. Meanwhile, the high-risk group had a worse prognosis than the low risk group. The expression status of ten genes in clinical samples was presented in Figure 4A (bottom). Among the ten genes, UBE2C and CDC20 are highly expressed in shorter survival time samples with the significant difference. The 1-, 3- and 5- year AUC (ROC) value of PRCC patients in the risk group were 0.926, 0.814, and 0.755, respectively (Figure 4C), which can further support the predictive effectiveness of the risk model.

### Identification of the expression, alteration frequency, and prognostic value of these genes in PRCC

**Figure 5:**
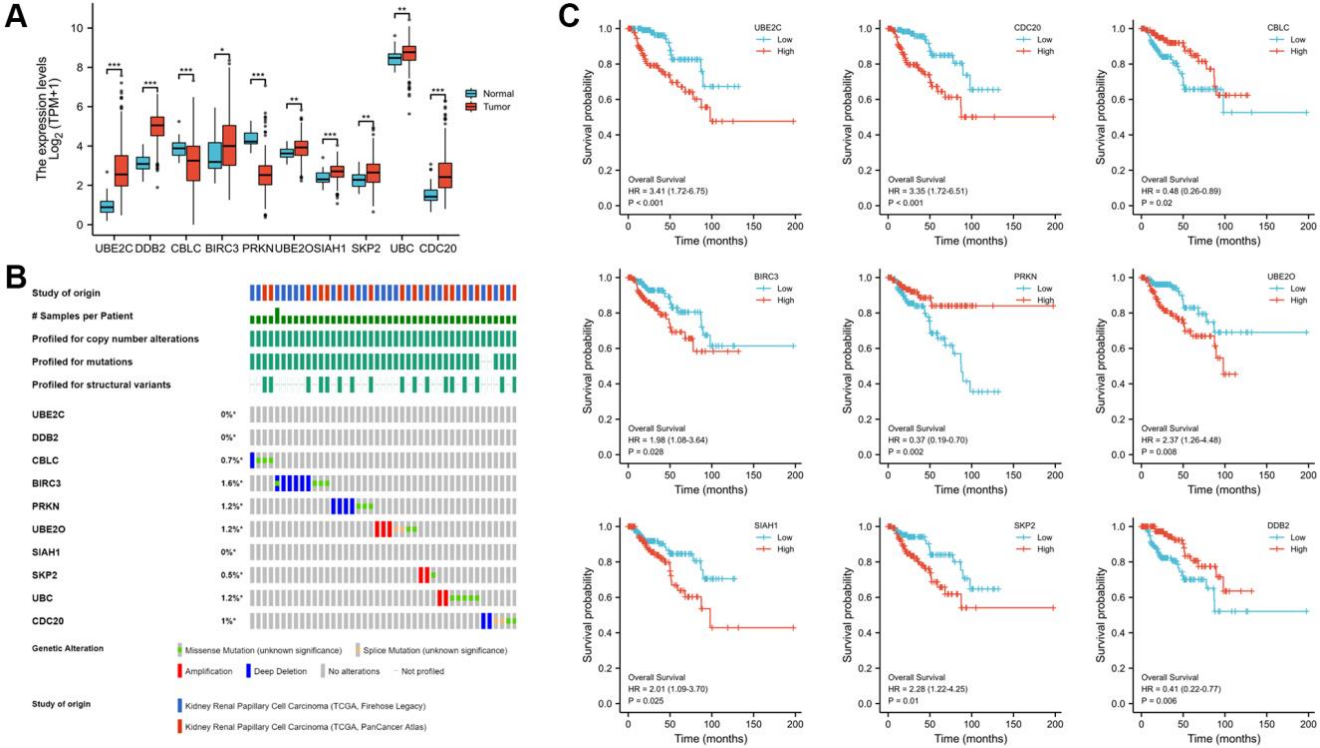
**Expression, alteration frequency, and prognosis of the 10 risk genes.** (**A**) Validation of the mRNA expression levels of the ten risk genes in PRCC tissues and normal renal tissues. (**B**) Validate signature genes by the blast with independent cases via the cBioportal database. (**C**) Association between gene expression and overall survival in PRCC evaluated using the Kaplan-Meier plotter database. Probe number, hazard ratio (HR), and log-rank *P* values are shown in each panel. ^*^P < 0.05, ^**^P < 0.01, ^***^P < 0.001.

The Human Protein Atlas was utilized to analyze the protein expression of the ten hub genes to confirm the mRNA expression consequence. All genes were extensively expressed in PRCC except CBLC and PRKN (*P* < 0.001, Figure 5A). The cbioportal database's analysis of genomic mutation in 10 genes was also conducted. Among the 576 PRCC samples analyzed, genetic mutations of these risk genes were identified in 43 cases (7.4%, Figure 5B). More interesting, there were no mutation in UBE2C and DDB2. The result may suggest that UBE2C and DDB2 do not have genetic mutations, but have protein or post- transcriptional level changes. Utilizing the Kaplan- Meier plotter obtained from the cBioportal database, the associations between the expression of these genes and clinical outcomes were subsequently assessed. High expression of UBE2C, CDC20, BIRC3, UBE2O, SLAH1, SKP2 and UBC was noticeably related to a worse prognosis, whereas CBLC, PRKN, DDB2 correlated with a better prognosis. Nine of the key genes were shown in Figure 5C (all *P* < 0.05).

### Correlation of the risk genes and PPIN construction

**Figure 6:**
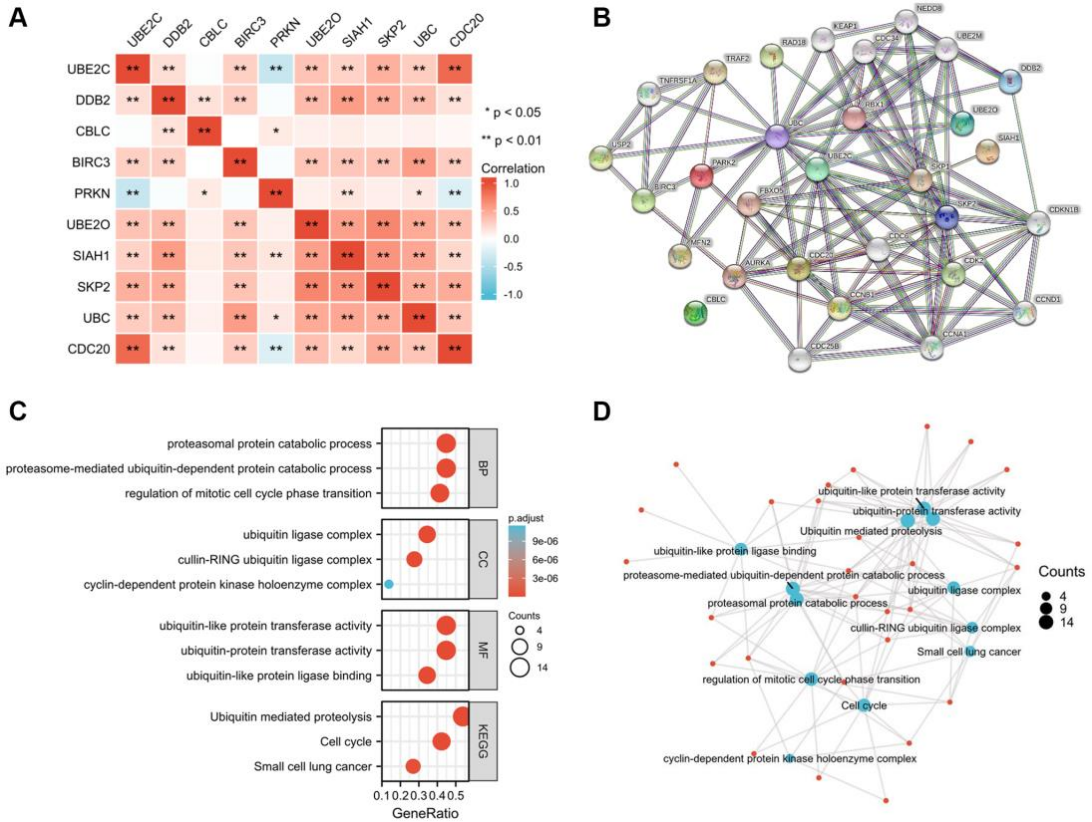
**Hub-related gene enrichment analysis.** (**A**) The heatmap shows the Pearson correlation between the risk genes in PRCC. (**B**) The top 20 genes that correlated with those risk genes were obtained through the STRING tool. (**C**) GO (BP, CC, and MF) and KEGG pathway analysis of 10 hub genes and 20 interacted genes. (**D**) Net-plot of these gene enrichment analyses. “Red” represents the positive correlation and “Blue” represents the negative correlation. Abbreviations: GO: gene ontology; BP: biological process; CC: cellular component; MF: molecular function; KEGG: Kyoto encyclopedia of genes and genomes. ^*^P < 0.05, ^**^P < 0.01, ^***^P < 0.001.

To determine the genetic interactions between these genes, the Pearson correlation coefficient calculations were performed. Besides CBLC and PRKN, other eight genes were found to have favorable correlations with one another by regression analysis (Figure 6A). The Protein-Protein Interaction Networks (PPIN), which include 10 hub genes and 20 interacted genes, is displayed in the STRING database (hub-interaction genes). GO and KEGG pathway analyses revealed that these genes were functionally enriched in the regulation of important processes such as the mitotic cell cycle phase transition and proteasome-mediated ubiquitin-dependent protein catabolism (Figure 6B–6D).

### Association of the risk genes expression and clinicopathological characteristics of PRCC

**Figure 7:**
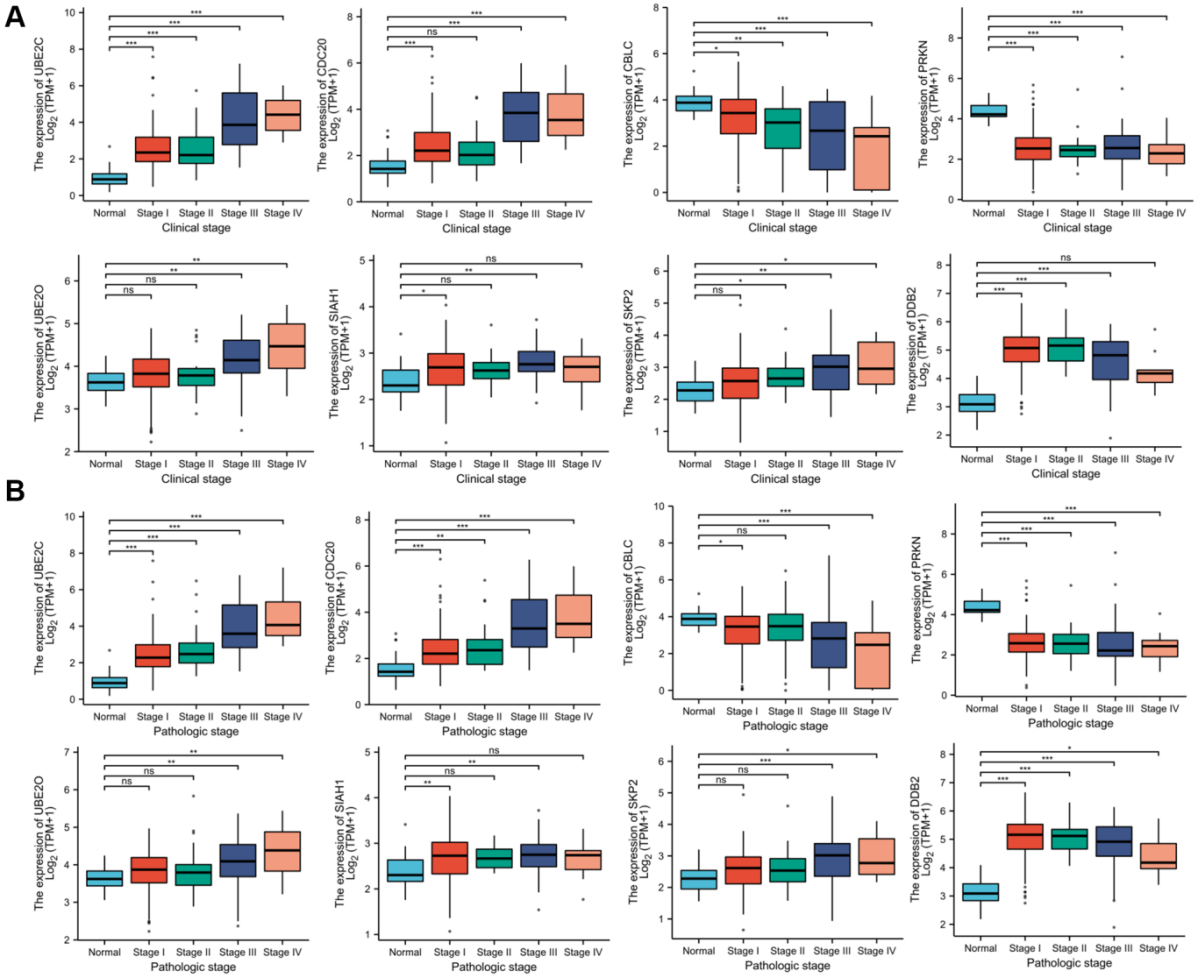
**Correlation between risk genes expression and clinicopathological parameter of PRCC.** (**A**) Boxplot showing that superior expression of UBE2C, DCB2, UBE2O, SIAH1, SKP2, and CDC20 were significantly related to advanced pathological grades, whereas low expression of CBLC and PRKN was significantly correlated with severe pathological features. (**B**) Boxplot showing that the expression of UBE2C, DCB2, SKP2, UBE2O, SIAH1, and CDC20 mRNA in PRCC samples are significantly correlated with severe pathologic staging and the expression of CBLC and PRKN at mRNA level were lower in patients with stage 4. ^*^P < 0.05, ^**^P < 0.01, ^***^P < 0.001, Abbreviations: ns: non-significance.

Since these hub genes were found to be altered in pan cancer, the present study attempted to explore the potential oncogenic role of the hub genes in PRCC [21, 22]. The TCGA-PRCC datasets were investigated for evidence of a relationship between gene expression and the clinicopathological status of patients with PRCC. Statistical analysis of the TCGA-PRCC cohort indicated that the expression of CBLC and PRKN was positively linked with favorable clinical staging. However, high expression of UBE2C, DCB2, UBE2O, SIAH1, SKP2, and CDC20 in PRCC tissues was associated with worse clinical and pathological stages (Figure 7A and 7B).

We then evaluated the association of the selected genes with the clinical features of patients in the TCGA-PRCC cohort subgroups. UBE2C was found to be strongly positively connected with aggressive phenotypes including nodal metastases, advanced clinical cancer stage and tumor grade by evaluating the PRCC cohort (Supplementary Figure 1A–1D). Grouped by gender (*P* < 0.001), tumor grade (*P* < 0.001), and race (*P* < 0.001), high expression of UBE2C was linked to shorter overall survival (Supplementary Figure 2A–2D). Recent studies discovered that linear ubiquitination is critical to innate and adaptive immune signaling [23]. We also explored the correlation between UBE2C and checkpoint inhibitors in PRCC (Supplementary Figure 3A). The coefficient and *p*-value of UBE2C and CDC20 are significant (Supplementary Figure 3B and 3C) [24].

### Protein expression of UBE2C and CDC20

**Figure 8:**
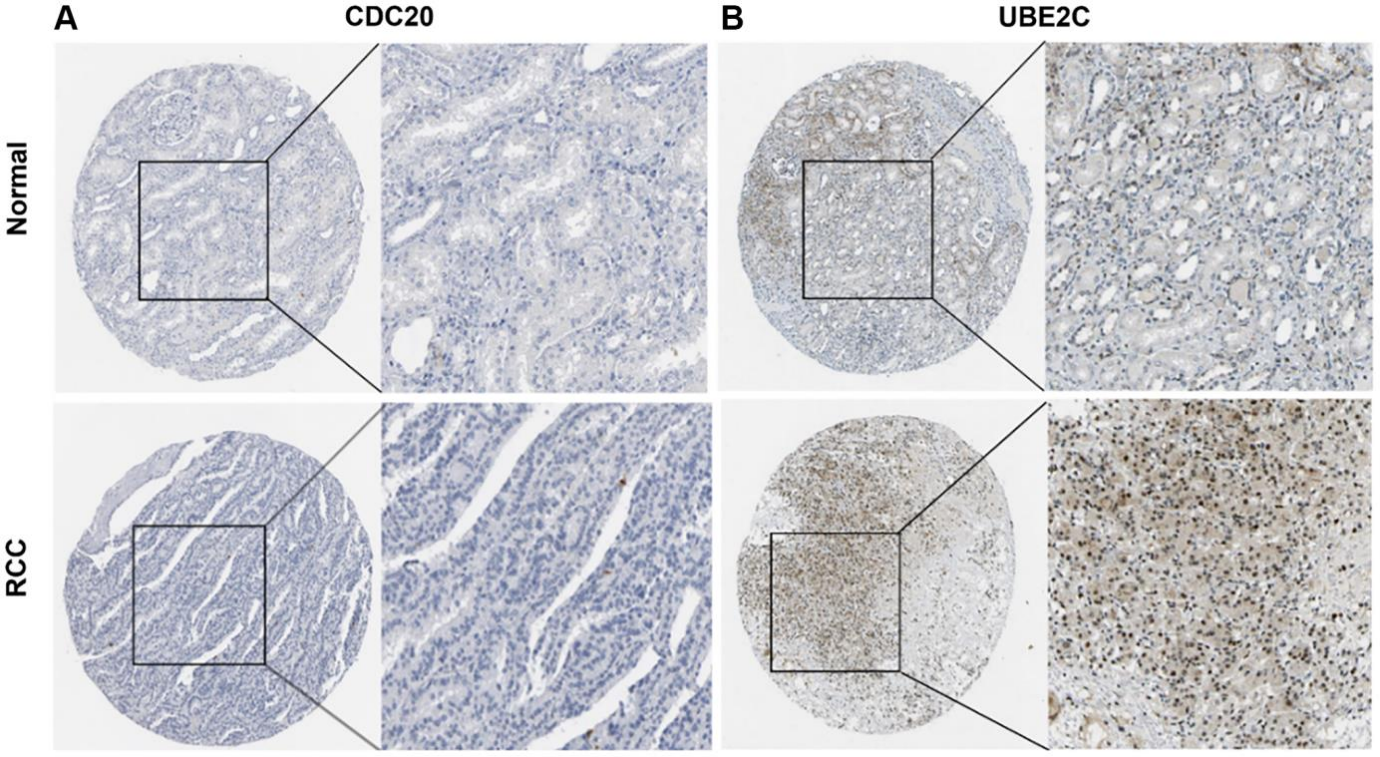
**Representative immunohistochemistry images of risk genes in PRCC and non-cancerous renal tissues derived from the HPA database.** (**A**) Validation of CDC20 by the human protein atlas database. (**B**) Validation of UBE2C by the human protein atlas database. Abbreviations: PRCC: papillary renal cell carcinoma; HPA: Human Protein Atlas.

The HPA database was utilized to investigate the levels of UBE2C and CDC20 protein expression in PRCC because the two genes directly correlated with clinicopathological characteristics and patient survival. In normal renal tissues only modest levels of UBE2C protein expression were observed, but in renal cancer tissues medium and high levels of UBE2C expression were detected. However, modest levels of CDC20 protein expression were found in both normal and renal cancer tissues (Figure 8), indicating that the transcriptional and translational levels of UBE2C expression in PRCC were not differential.

### Correlation of risk genes expression and tumor-infiltrating immune cells

Tumor-infiltrating lymphocytes (TILs), are a key indicator for the prognosis and effectiveness of immune therapy [25]. To understand CDC20 and UBE2C in the regulation of immune infiltration, we discussed the correlation between both gene expressions and immunological markers in PRCC. As showing in Figure 1A, UBE2C was adversely connected with the number of TILs, but CDC20 was strongly correlated. Particularly, the expression of CDC20 and UBE2C was inversely connected with the quantity of pDC (r = −0.136, *P* = 0.0207) and iDC (r = −0.219, *P* = 0.000169) and favorably correlated with the abundance of Act CD4 T cells (r = 0.451, *P* = 2.2e-16; r = 0.443, *P* = 2.2e-16).

### Relationship between risk genes expression with immunoinhibitors

Immune checkpoint inhibitors (ICIs), remarkably innovative immunotherapy for the treatment of various types of cancers, have already gradually improved patient outcomes. We examined the relationship between gene expression of CDC20 and UBE2C and 24 immunoinhibitors among pan cancer to evaluate their clinical significance in association with immune moderation. The heat map of immunoinhibitors, in particular, demonstrated the favorable correlations with CDC20 and UBE2C in PRCC (Figure 1), as well as IDO1 (r = 0.363, *P* = 2.34e10; r = 0.385, *P* = 1,47e11) and CD274 (PD-L1, r = −0.112, *P* = 0.0562; r = −0.182, *P* = 0.0018). Due to the critical role of PD-L1 in immunotherapy, CDC20 and UBE2C can serve as a biomarker for anticipating ICB response. Together, effects on TILs and immunoinhibitors suggested that CDC20 and UBE2C may be critical in controlling the immunological infiltrates in PRCC.

### UBE2C can affect the proliferation, migration, and immune tolerance potential of cancer cells *in vitro*

**Figure 9:**
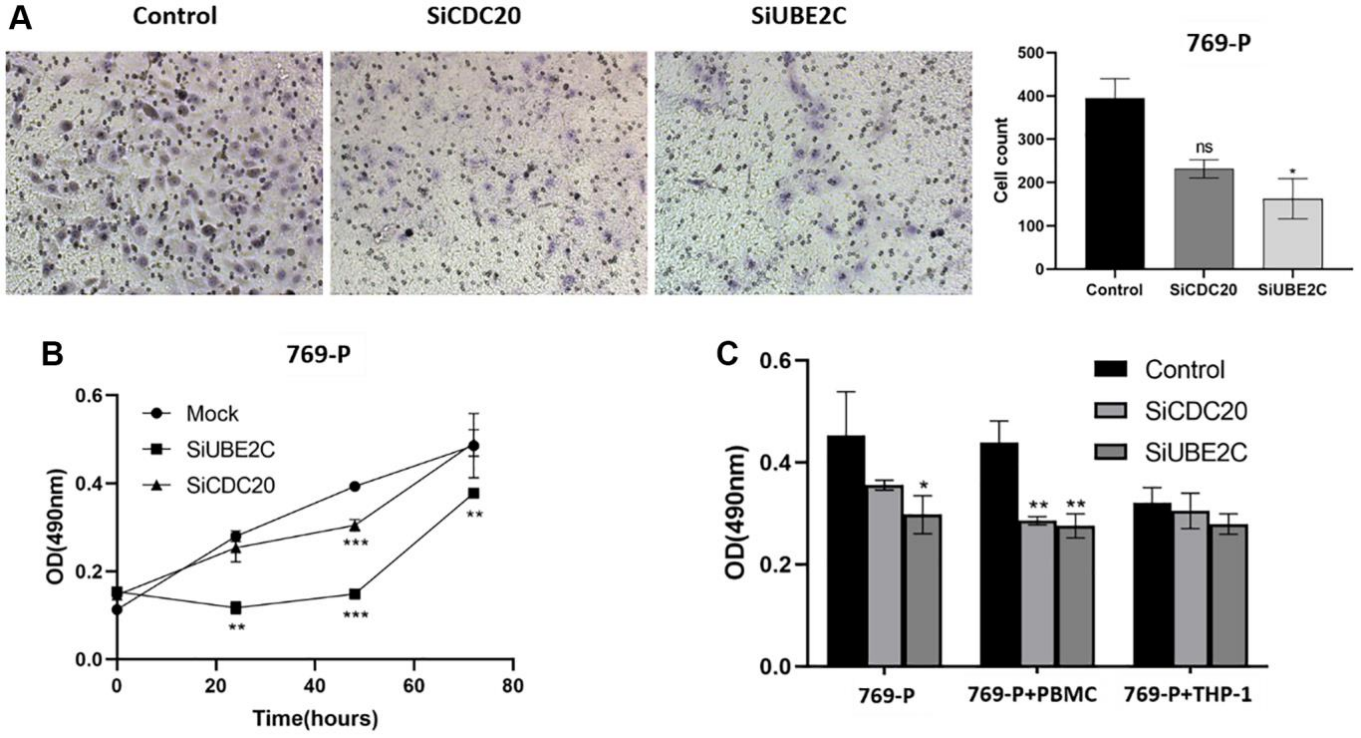
**Downregulation of UBE2C and CDC20 inhibited the proliferation, migration, and immune tolerance in 769-P cells.** (**A**) Representative images and quantitative numbers of migrated cell across the membrane porous over 48 hours were determined. (**B**) Cell proliferation of 769-P cells with siRNA of CDC20 and UBE2C was detected with CCK8 assay. (**C**) MTT assay was performed to evaluate the effect of CDC20 and UBE2C knockdown on 769-P cells proliferation after co-cultured with PBMC and THP-1. Mean ± SEM; ^*^P < 0.05, ^**^P < 0.01, ^***^P < 0.001. Abbreviation: ns: non-significance. All experiments were repeated three times.

We used the siRNA technique to downregulate CDC20 and UBE2C in two renal cancer cell lines to investigate their role in renal carcinoma. *In vitro* transwell assays showed that the proliferation of 769-P cells was reduced by UBE2C knockdown compared to vector control (Figure 9A). UBE2C knockdown prevented cell proliferation analyzed by using the MTT test (Figure 9B). The anti-immune effect on 769-P cells was also calculated by it after co-culturing with immune cells. When co-cultured with PBMC (peripheral blood mononuclear cell) cells, as showed in Figure 9C, CDC20 and UBE2C knockdown prevented the proliferation of the 769-P cells, but there was no significant connection with THP-1 (human monocytes). These data showed that UBE2C knockdown affected the proliferation, migration, and immunological tolerance of 769-P cells.

## DISCUSSION

The ubiquitin system is complicated, and multifaceted, and is crucial for the modulation of a vast number of cellular processes. The ubiquitin-proteasome system (UPS) directs protein degradation at the epigenetic, genomic, or post-translational levels through the proteasome, and regulates a wide array of cellular processes as well as key suppressors or oncoproteins in select tumors. Previous studies have noted that UPS genes be involved in the regulation of pathogenesis and progression of various types of tumors [26, 27]. The F-box protein Skp2, a component of RING E3 ubiquitin ligases, was significantly correlated with several cancer-associated signaling pathways, including proliferation, cell cycle progression, migration, and invasion of cancer cells [28]. U-box-type E3 ubiquitin ligase PPIL2 can inhibit EMT and invasion of breast cancer by interacting with the classical EMT transcription factor, SNAI1, to enhance its ubiquitin-dependent degradation [26]. Ubiquitin-conjugating enzyme E2H (UBE2H) can regulate the EMT program and metastasis in LUAD [27]. Akinobu et al. reported that FBXW7, a well-studied F-box protein, can affect the pathological development of cancer by degrading the Notch pathway and being a useful diagnostic marker for cancers [29]. These studies suggest that the gene signature in the regulation of UPS demonstrates marked clinical availability.

However, the effect of a single gene on the tumor is fairly limited, and the predicating role for the prognosis of patients is not reliable. Besides, few studies have addressed prognostic and predictive evaluation biomarkers for immunotherapy utilizing UPS genes. To further understand of mechanisms underlying UPS expression in cancer, we concentrated on the expressive features and prognostic value of UPS genes and finally constructed a risk model. Based on our findings in this study, UPS genes are highly enriched in PRCC compared to normal tissues. In our study, we obtained 10 genes from 140 genes involved in UPS that was significantly related to PRCC as prognostic factors via Lasso regression analyses. Subsequently, a prognostic signature constructed using these 10 hub genes was utilized to better distinguish outcomes in PRCC with distinctive clinical features and genetic alterations. KEGG pathway enrichment analysis based on 10 hub genes and 20 interacted genes was enriched in the cell cycle pathway and ubiquitin-mediated proteolysis. In accordance with cellular functions, UBE2C and CDC20 displayed a dynamic increase from pre-PRCC status to PRCC, suggesting that UBE2C and CDC20 might be key driver genes for PRCC progression through enhanced aggressive behaviors of PRCC cells with overexpression of both genes. Instead, founded on the observation in this TCGA dataset, UBE2C and CDC20 might serve as hub genes that participate in tumorigenesis and PRCC progression. We further explored the potential function of UBE2C and CDC20 in PRCC. In renal cell carcinoma (RCC), the topmost two signaling pathways affected by UBE2C are cell cycle and DNA replication pathways. Knockdown of UBE2C significantly inhibited proliferation and migration in renal cancer 786-O cells *in vitro*, confirming our bioinformatics analyses.

These results seem to be compatible with extra research which found that UBE2C, CDC20, and other key genes within this prognostic signature are related to the occurrence and development of tumors. UBE2C encodes a member of the E2 ubiquitin-conjugating enzyme family and participates in the regulation of the expression and activity of the mTOR/PI3K/AKT pathway [30]. UBE2C is overexpressed in various types of cancer including cervical cancer, breast cancer and non-small cell lung cancer (NSCLC) participates in the progression of these tumors [21, 31]. CDC20 is a crucial target of the spindle assembly checkpoint and can activate the anaphase-promoting complex/cyclosome (APC/C) [32]. Aberrant expression of CDC20 is associated with malignant progression and poor prognosis in various types of cancer, including gastric, urothelial bladder cancer, oral squamous cell carcinoma, and hormone-positive breast cancer [33–36]. One study by Yongwen (2019) identified 9 key genes in clear cell renal cell carcinoma, which including UBE2C and CDC20 [37]. Detailed information of the investigation showed that both of UBE2C and CDC20 were clinically independent prognostic factor for ccRCC patients. Taken together, these studies support the notion that UBE2C and CDC20 are hub genes in different types of renal cancer. Apart from that, UBE2C and CDC20 can also be found in other cancers [38]. Although the mechanisms of the hub genes in PRCC were not examined, the carcinogenesis of these hub genes can indicate from the protein-protein interaction (PPI) and the gene enrichment analysis.

The tumor microenvironment plays a vital role in the development of tumors, especially in the immune infiltration of tumor cells [39]. This not only influences tumor formation but also the efficacy of ICIs [40]. Significant correlations between two hub genes and immune-related genes were found.

CDC20 and UBE2C have been found to be increased Act CD4 T cell infiltration but less extensive infiltration of pDC and iDC cells. Poor clinical outcomes for patients and immunosuppression status have been proven be correlated with Act CD4 T cell infiltration levels [41, 42]. At both the transcriptional and protein levels, the quantity of DCs is positively connected with T cell infiltration in human neuroblastoma and is associated with a good prognosis [43]. Considering that immune cell infiltration is an important factor in predicting the treatment effect of ICIs, we intend to investigate the predictive value of the hub genes in PRCC. Additionally, our research revealed a weak link between the expression of PD-L1 and UBE2C and CDC20. The key to immunotherapy relied on immune cell infiltration, and PD-L1 expression was no longer a useful biomarker for immunotherapy. Constructed signature that can predict the efficacy of ICIs may be ascribed to the fact that the signature was related to immune cells rather than PD-L1 expression.

## CONCLUSION

In summary, this study has presented the subsequent contributions to PRCC research. First, a prognostic signature based on UPS regulator-related genes is established, and validated its applicability via several methods. This signature can be utilized to effectively evaluate the prognoses of PRCC patients and may also potentially predict responses to ICI treatments. Second, UBE2C and CDC20 within this signature may participate in the progression of PRCC and act as potential therapeutic targets. Certain shortcomings and deficiencies in this study remain. To begin with, the establishment and validation of the signature were based on the public sequence data. Further validation, such as prospective studies and clinical trials of PRCC patients in multi-centers, might make the signature more convincing. Although we test some functions of these key genes in experimental assays, the precise regulatory mechanism of these genes in PRCC remains to be clarified.

## Supplementary Materials

Supplementary Figures
