# Supplementary material for: Identification and validation of ubiquitin-proteasome system related genes as a prognostic signature for papillary renal cell carcinoma
Source: Aging (Albany NY). 2022 Nov 16;14(23):9599–616. doi: 10.18632/aging.204383 (PMC9792205; doi:10.18632/aging.204383)
Supplement: Supplementary Figures [file aging-14-204383-s001.pdf]

SUPPLEMENTARY FIGURES

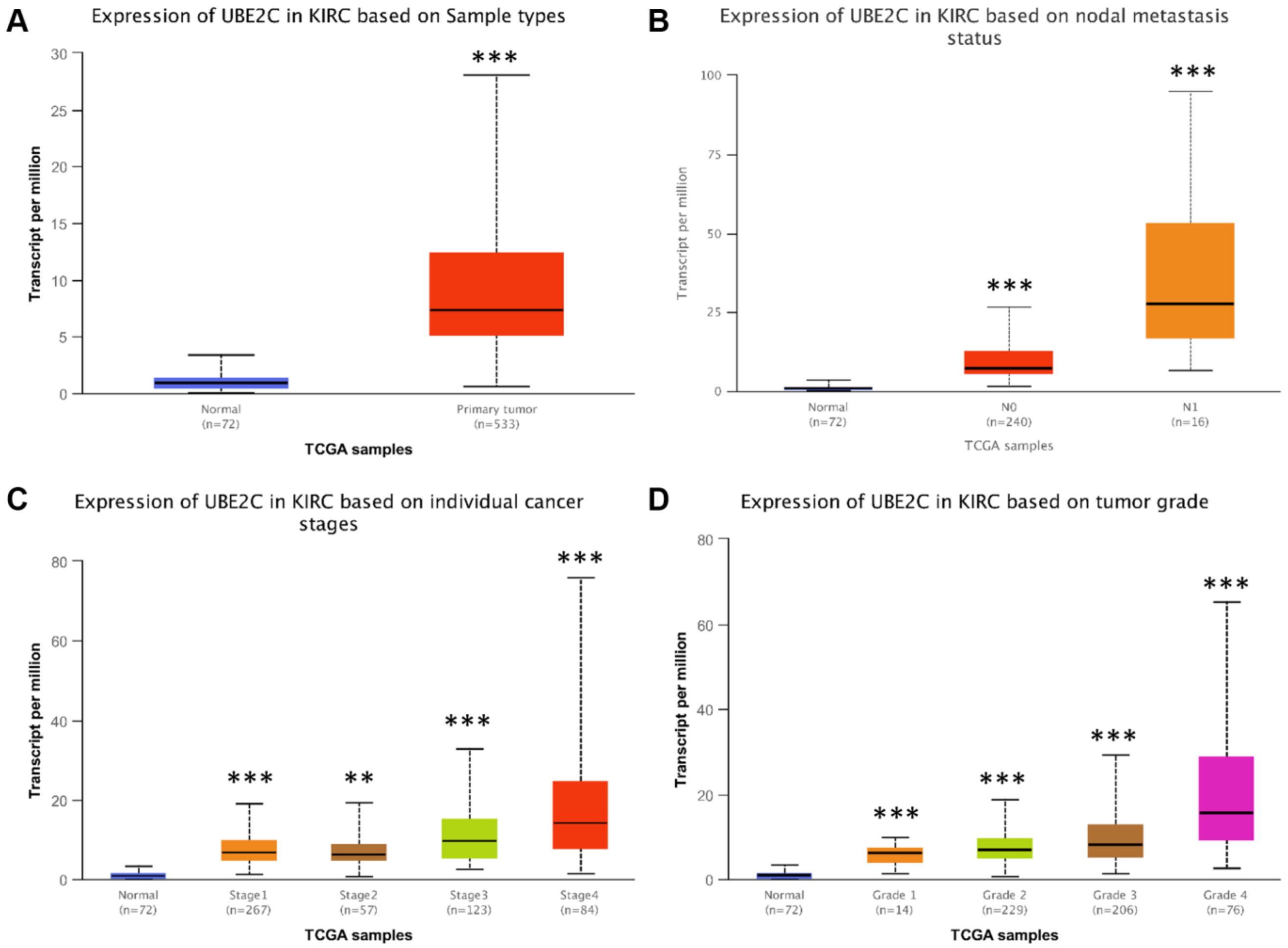

Supplementary Figure 1. Association of UBE2C mRNA expression with clinicopathologic characteristics. (**A**) sample types. (**B**) nodal metastasis status. (**C**) individual cancer stages. (**D**) tumor grade.

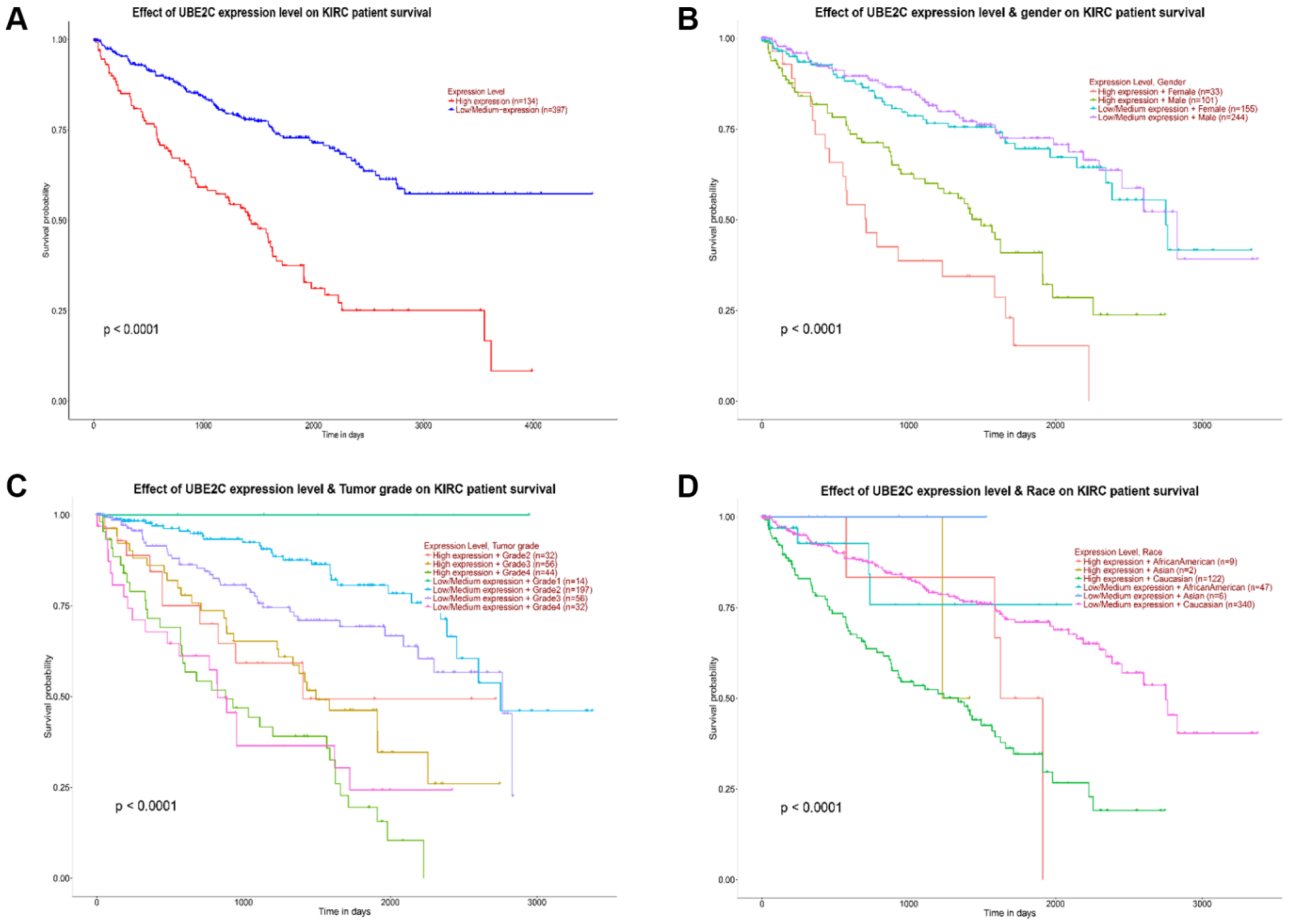

Supplementary Figure 2. The prognostic value of mRNA levels of UBE2C factor and subclinical factors in ccRCC patients. (**A**) The overall survival of UBE2C in PRCC for all cases ($P < 0.0001$). (**B**) The overall survival of UBE2C and gender in PRCC ($P < 0.0001$). (**C**) The overall survival of UBE2C and tumor grade in PRCC ($P < 0.0001$). (**D**) The overall survival of UBE2C and race in PRCC ($P < 0.0001$).

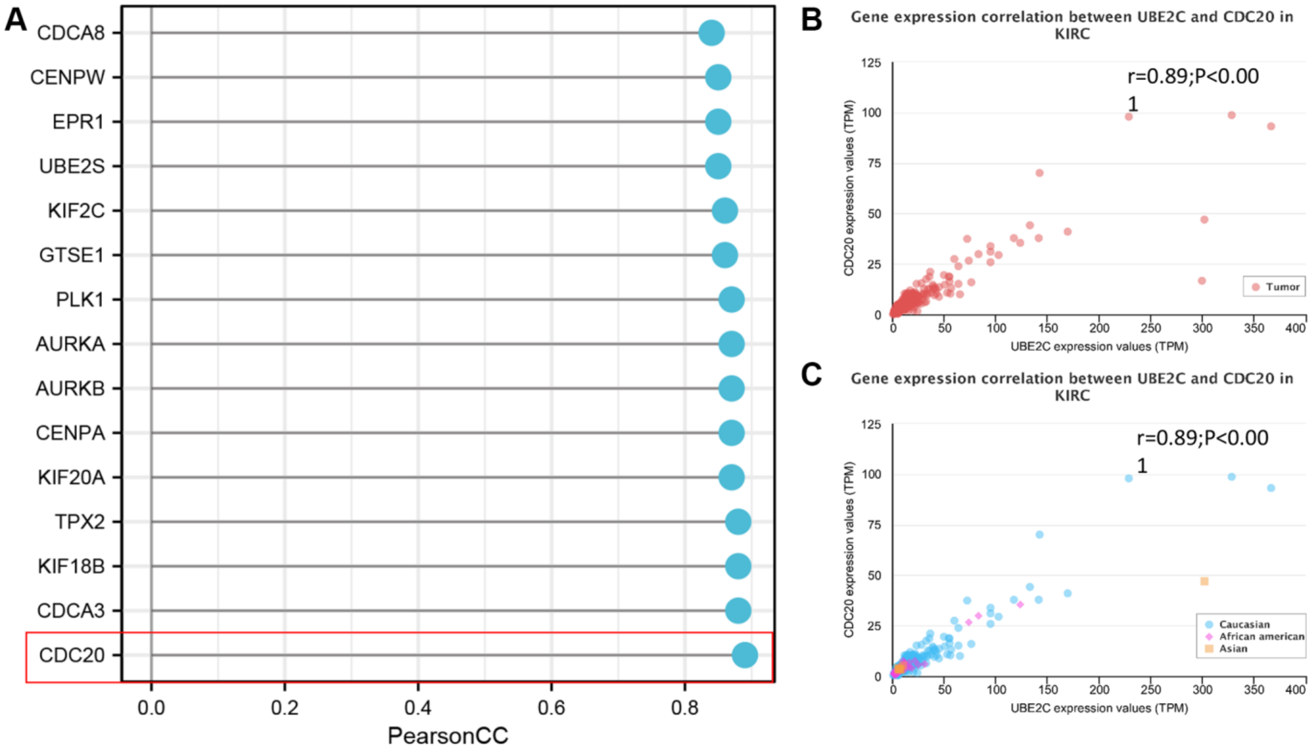

Supplementary Figure 3. The relationship of UBE2C with checkpoint inhibitors in PRCC. (**A**) The Pearson correlation of UBE2C with 47 different checkpoint inhibitors and 15 of them was displayed. (**B** and **C**) The coefficient and *p*-value of UBE2C and CDC20 were shown on the graph.
